# Psychology and the fall of Communism: The special case of (East) Germany

**DOI:** 10.1002/jhbs.22238

**Published:** 2022-11-29

**Authors:** Mitchell G. Ash

**Affiliations:** ^1^ Department of History University of Vienna Vienna Austria

**Keywords:** East Germany, German unification, history of psychology, reflexivity, transitology

## Abstract

The fall of Communism is now universally agreed to be what the philosopher Hegel called a world historical event—one that few predicted but nearly everyone saw as inevitable after it happened. In the aftermath many lives—and worldviews—changed, not only, but also in the human sciences. These remarks attempt to address in a preliminary way both the impact of the fall of Communism on psychology in former East Germany (including changes in personnel and approach) and the ways in which these sciences were employed as resources for reflection on the Communist past as well as the transition to new social and political regimes.

## 1 INTRODUCTION

The fall of Communism is now universally agreed to be what the philosopher Hegel called a world historical event—one that few predicted but nearly everyone saw as inevitable after it happened. In the aftermath, many lives, careers, and worldviews changed, not only, but also in the human sciences. In the following remarks, after a very brief summary of the political and science policy contexts, I will attempt to address in a preliminary way the impact of the fall of Communism on psychology in the former German Democratic Republic (GDR), or East Germany. In doing so I will address changes in personnel, but will also discuss the ways in which psychological concepts or metaphors were employed as resources for reflection on the Communist past as well as the transformations that resulted from the fall of Communism.

One might well ask: Why East Germany? After all, this is an atypical, indeed a unique case. In all of the other Eastern European states outside the Soviet Union, Communist rule was ended while the nation‐states largely remained in place, with two exceptions: Czechoslovakia, which peacefully split into two nations in 1992, and Yugoslavia (a socialist regime that had been independent of the Soviet bloc), which divided violently into multiple nation‐states. However, only the GDR—to be precise, the first freely elected government of that state—decided to exercise the option, made available by Article 23 of the West German Basic Law, of joining the existing Federal Republic of Germany. Nonetheless, many of the processes at work in the case of East Germany also took place in other former Communist countries during and after the fall of Communism, including massive, though mainly temporary, political engagement by nonpolitical actors, and reflection—or refusal to reflect—upon the successes and failures of the fallen regimes.

The approach I adopt here comes from a larger project in which I am currently engaged, with the ambitious working title “Scientific Changes in Times of Political Upheaval: Germany (and Austria) 1918, 1933/38, 1945, 1990” (For early statements, see Ash, 1999, 2004). In this project, case studies from individual disciplines are considered not for themselves, but as examples of larger historical processes. Speaking very broadly, I regard scientific changes in times of political upheaval as *reconfigurations of resource constellations*. In this approach, resources are not limited to funding alone, but also include personnel (now often called “human resources”), institutional power, ideological (re)codifications of sciences, scientific research practices and tools, and last not least reflexivity—the utilization of one's own experiences as a resource for the development of new scientific research programs (Ash, 2016).

The paper has three parts. After a very brief sketch of the political and policy contexts, I consider, again in a preliminary way, the impact of German unification on psychology as an academic discipline in what came to be called the “new German states” (*Neue Bundesländer*). The focus here is on personnel, institutional resources such as the number and content of professorships, and curricula. In part three, I then consider two examples of the utilization of psychology as a resource for reflection on the Communist past and on the transformations of the early to mid‐1990s.

## 2 PART ONE—THE POLITICAL (AND SCIENCE POLICY) CONTEXTS IN OUTLINE

Although the failure of the East German regime to provide for its citizens had become obvious by the 1980s, and in the summer and autumn of 1989, thousands had begun to escape through the newly opened Hungarian border or to demonstrate on the streets in Leipzig, Berlin, and other cities, NO ONE expected the fall of the Berlin wall on November 9, 1989. A few observers said at the time that the opening of the border meant the end of the GDR because its planned economy was not sustainable without a sealed border, but many others still hoped for a reformed socialist state. Even the West German leadership initially expected that unification would take between 5 and 10 years (Ritter, 2007). As we know, that time frame shrank with increasing speed. “The Rush to German Unity” (Jarausch, 1994) happened in stages, with at least four decision points, not all of them West German: the offer of an social, economic and currency union made by the government of Helmut Kohl in mid‐February 1990; the elections in East Germany in March and the statement of the new East German government in April in favor of unification by joining the Federal Republic, rather than the two states' agreeing on a new constitution first; the collapse of the East German economy, beginning even before the state treaty establishing the currency union was signed in mid‐May; and finally, the decision by the Federal government in Bonn to accelerate negotiations for a “second state treaty” after the first meeting of the parties in early July.^1^ Until that final decision point, NO ONE had imagined that unification would be agreed in mid‐August and actually occur on October 3.

This accelerating time frame is important for understanding how science and higher education policy evolved in the same period (for the following in detail, see Ash, 2020; Part II). In higher education, the “reform socialist” Modrow government took a first step toward reform by closing the Institutes for Marxism‐Leninism at the universities and ending required studies in that field in early 1990. This appeared to establish some credibility for the claim that an independent GDR could reform itself. In the Academy of Sciences and the universities, however, internal power struggles dominated the scene (On the Academy of Sciences, see inter alia Mayntz, 1994; and Stark, 1997; on the universities, see e.g., Jarausch, 2012 and Pasternak, 1999). Apparently, nearly everyone on the East German side assumed until May 1990 or even later that the GDR would continue to exist for some time. It thus came as a complete surprise to many (including the recently elected President of the GDR Academy of Sciences, who was present) when the decision was announced at a joint press conference on July 3, 1990, in Bonn that representatives of both German governments had agreed, in essence, (1) to extend the West German institutional framework for the support of science and higher education to the East, and (2) to ask the West German Science Council (*Wissenschaftsrat*) to evaluate the research institutions of the Academy of Sciences of the GDR with the aim of “adapting” them to the West German system, and also separately to evaluate higher education structures in the new German states. The separation of the Academy of Sciences of the GDR as a learned society from its vast association of research institutes and the dissolution of the latter, negotiated after this meeting and formulated in Article 38 of the Unification Treaty of September 1990, was the most important science policy provision in that document.

In higher education, change already began under the Modrow government when required instruction in “Marxism‐Leninism” was ended in January 1990, but this did not automatically lead to the closure of the institutes where that subject had been taught. The radical transformation of East German higher education that began after unification took place in four phases (for recent overviews see Ash, 2021; Blecher & John, 2021): (1) the “wrapup” (*Abwicklung*) and “refounding” of selected academic disciplines deemed ideologically corrupted, including philosophy, history, pedagogy, and what might be called the societal sciences (*Gesellschaftswissenschaften*) based on a questionable reading of the Unification Treaty (see section two below); (2) passage of so‐called higher education “renewal” legislation in the new East German states beginning in July 1991, mandating the temporary suspension of university self‐governance and a distinction between “professors under previous law” and “professors under new law,” meaning that teaching staff had to reapply for their positions and undergo political and academic evaluation before being rehired, and the evaluation of all teaching staff according to “political and moral integrity” with dismissal being the norm for those who had held high‐level posts in the Socialist Unity Party or had collaborated with the Secret State Police (Stasi); (3) parallel to this a full‐scale “restructuring” in higher education, including the founding of new universities, and—perhaps most important—the restructuring of existing universities and teaching staffs according to West German norms, as well as the introduction of new curricula on West German models; (4) serious budget cuts from 1992 onward, leading to still further staff reductions. Taken together, all of this amounted to a one‐sided revolution from above, generally labeled “renewal” at the time by its West and East German advocates.

Many of those affected by these policies felt betrayed. Very soon, the claim that unification had been perverted from an agreed arrangement by two states into a “colonization” or “takeover” of the East by the West became a widespread narrative accepted by many in the East, which persists to this day (Kowalczuk, 2019). Before we accept this narrative, we must remember four things:

(1) The East German population was itself divided. Some groups hoped to the end for a “third way” between state socialism and capitalism, while the majority sought rapid unification to maintain political stability and achieve rapid economic prosperity with the help of West Germany's strong D‐Mark, only to see their hopes dashed and many of their jobs disappear due to the collapse of the East German economy. For the vocal minority who took part in the citizens' revolt that actually brought down the East German regime, the victory of the CDU‐led “Alliance for Germany” in the elections of March 1990 ended their hopes and was therefore also seen by many as a “takeover,” even though the new East German leader, Lothar de Maizière, was East German.
(2) In contrast. very few scientists and scholars had actively participated in the peaceful revolution of 1989. The majority of senior scholars and scientists continued to have a vested interest in maintaining existing East German institutions and their own positions in them, against the opposition of mid‐level academic staff and some dissident students.
(3) Not only the provisions of the Unification Treaty itself, but all of the policy arrangements enacted thereafter were the joint work of East and West Germans, meaning that many East Germans in leading positions, most prominent among them Hans Joachim Meyer (CDU), East Germany's last minister of education and later first minister of science and art in the “free state” of Saxony, did not share the vested interests just mentioned, and were not among the turncoats (*Wendehälse*) who opportunistically changed sides later.
(4) Even the head of the West German Science Council at the time, Dieter Simon (Director of the Max‐Planck‐Institute for Legal History), far from wanting to be a colonizer, assumed at the outset that the Unification Treaty had guaranteed the stability and staffing of the universities in the new East German states, and that they would soon receive new and better‐trained personnel from positively evaluated institutes of the Academy of Sciences (D. Simon, 1992). Again, NO ONE expected the actual outcome in advance.

Finally, it is time to ask: How did all of this impact psychology?

## 3 PART TWO: THE IMPACT OF THE FALL OF COMMUNISM ON PSYCHOLOGY—RECONFIGURING “HUMAN RESOURCES” AND INSTITUTIONAL CONSTELLATIONS^2^

Article 13 of the Unification Treaty set a deadline of 31 December 1990, by which date the new German state governments were required to decide whether or not state institutions were to be dissolved or continued. The relevant German word is *abwickeln*—a term taken from banking, originally meaning the orderly conclusion, or “wrapping up” of a business deal. Citing, but actually misapplying, this treaty provision, academic disciplines considered compromised by their involvement with the Communist regime, including law, the social sciences, pedagogy, philosophy, and history, were “*abgewickelt*” by order of the governments of the new German states. The professors and scholarly staff in these fields were dismissed, and the disciplines were then “refounded” with new staff, many of whom came from the West. The Unification Treaty actually mandated such decisions only for entire state institutions, or for parts of them that could operate independently. This might have applied to entire universities, but did not apply to university departments. When these decisions were actually challenged in administrative and labor law courts by the Senate of the Humboldt University and also by the faculty of history there, the challengers won. Elsewhere, state and university officials proceeded as though their actions were mandated by the Unification Treaty (for more detailed accounts see Ash, 1997, 2010, and the literature cited there).

These decisions were acts of *epistemic politics*. By this I mean the singling out of types of sciences as being ideologically corrupted by definition due to the roles assigned to them by the East German party and state, leading to policy decisions based on stereotypes that were themselves ideological in character, on which policymakers relied under perceived time pressure. In such circumstances, time is not a force of nature, but a matter of political choice. The widespread tale that the teaching staffs of East German universities were “Westgermanified” is true mainly for those disciplines that were on the *Abwicklung* lists in December 1990, but “Westgermanification” was not complete even in these fields. Humanistic fields like German studies or art history were also not on the lists, even though they too had been under party influence. Far more destructive in the succeeding years was a different kind of “westgermanification”—the “restructuring” of East German higher education institutions along the lines of West German personnel planning, which was not limited to any single type of discipline (see Ash, 2021 and the literature cited there).

Psychology and psychiatry were not on any such *Abwicklung* list. Were these human sciences exempted from dissolution, because experimental psychology had been considered a natural science in the GDR? Not quite: “Marxist‐Leninist” social psychology and pedagogical psychology were plainly not natural sciences, but rather “societal sciences” (*Gesellschaftswissenschaften*) in the GDR. Developmental psychology was taught in faculties of education, not natural science faculties, and the faculties of education at the universities were closed and then “refounded.” However, in Potsdam and Erfurt existing pedagogical academies were not closed, but folded into new universities. Thus, psychology's institutional situation post‐unification was hardly simple.

If we now look more closely at scientific personnel in psychology, two factors need to be considered: the personal, political, and scientific evaluation of individual scholars and scientists, which was legally mandated for all disciplines (see above), and the restructuring of psychology departments. Phases two and three sketched above were more relevant here than stage one (the *Abwicklungen*). During the rigorous evaluations of phase two, dismissals or forced retirements did take place in psychology. Most prominent among these was the case of experimental cognitive psychologist Friedhart Klix, longtime department head at the Humboldt University in Berlin and a scientist widely respected in the West, who worked with the Stasi in connection with his position and took early retirement to avoid dismissal. However, when we turn to decisions subsequently made about whom to appoint (or reappoint) to professorships in psychology departments, the picture changes. In the GDR there had been only four psychology departments, each highly specialized, with a single or at most two chairholders at the top: the Humboldt University of Berlin (Friedhart Klix, experimental psychology, information, and cognition), the University of Leipzig, formerly Karl‐Marx‐University (Manfred Vorwerg, social and pedagogical psychology), the Friedrich Schiller University in Jena (Hans Hiebsch, social psychology), and the Technical University in Dresden (Winfried Hacker, labor and industrial psychology). By the 1980s, this formal structure no longer reflected the actual power relations within the departments; the situation in Leipzig and Jena was less clearly dominated by a single chair‐holder than in Berlin and Dresden. Nonetheless, of the former institute heads only Hacker was retained, for reasons that are not entirely clear; Hiebsch died in 1990. Following unification, new departments were established both at older universities, such as Halle and Greifswald, and in newly founded ones, such as Potsdam. At the same time, curricula and personnel plans based on West German models were instituted by the new German states on the advice and recommendation of the German Psychological Society (Bredenkamp, 1993).

Alternative concepts were available; some saw the opportunity to give relatively new, in part socially and culturally critical fields like peace and conflict research, women's issues, migration/intercultural studies, or ecology and environmental protection, and above all political psychology, a chance, but the East German members of the “Structure and Appointments Commissions” soon lost the energy they would have needed to push them through over conservative West German opposition (Eckardt, 2019). Because departments now needed to offer instruction in all major subfields of psychology, including experimental, developmental, social and clinical psychology, professors needed to be appointed in fields that had not been emphasized locally before. How did all this impact personnel constellations in psychology?

A mix of East and West Germans appears to have been appointed in all East German psychology departments. Unfortunately, a survey showing how this mixture looked at each department is still lacking. Precise statistics are available at present only for the Humboldt University, and these refer only to appointments to professorships made there by the end of 1994 (Raiser, 1994). Of the four full chairs (C4 professorships) established in psychology there, two went to West Germans and two to East Germans. Of seven tenured associate professorships filled at that time, five went to East and two to West Germans. Thus, nearly two thirds of professorial appointments in Berlin in this early period (63.6%) came from the East.

Continuity in this sense appears to have been even higher at the Technical University in Dresden, in part because of its international reputation in applied and industrial psychology, but also because Saxony's Higher Education Renewal Law mandated preference for existing chairholders (here, Winfried Hacker) if there was no political objection and their work had been positively evaluated. At the University of Jena, after the separate political and scientific evaluations of teaching and scientific staff, roughly one‐third were recommended for retention (Eckardt, 2019). Among those retained in Jena was a historian of psychology Georg Eckardt, who was appointed professor for the first time and later served as department head, but new positions were filled mainly by colleagues from the West. Unfortunately, the numbers for the department in Leipzig are unknown to me thus far. More comparative research is needed to make a more general statement about whether East German psychologists profited or suffered from reunification.

Did these personnel decisions impact research programs, and if so, how? A major factor here was the restructuring of East German psychology departments due to the introduction of West German curricula, just mentioned. This tended to support external appointments to the new positions, since qualified local candidates were less likely to be available; many, but not all, of these new appointees came from West Germany. Whether the resulting “mixing” (in German: *Durchmischung*) of upper‐level university staff in the 1990s led to changes in research programs is not entirely clear. East‐West contacts in psychology had already begun the intensify after the International Congress of Psychology was held in Leipzig in 1980. At the Humboldt University, where experimental and mathematical psychology had already been predominant, “old Humboldtians” such as Bodo Krause, a mathematician turned statistical psychologist, worked alongside “new Humboldtians” like Peter Frensch, a specialist in learning and cognition who had received his PhD in the United States in 1989 (100 Jahre Institut, 1994; see also Ebisch/Ash 2010). However, based on his experience in Jena, Georg Eckardt (2019) suggests that “mixing” (*Durchmischung*) may not be the best term to use. He reports that West and East German professors worked collegially alongside one another, but pursued their research agendas separately, with relatively little collaboration.

## 4 PART THREE: PSYCHOLOGY AS A DISCURSIVE RESOURCE FOR REFLECTION ON THE COMMUNIST PAST AND ON THE TRANSITION TO A NEW REGIME

One of the truly amazing aspects of this history is the explosion of self‐examination among East Germans that began immediately before the fall of the Berlin wall. Psychologists participated in this trend in a number of ways: (1) academic work on the history of psychology in the GDR; (2) autobiographical reflections by East German psychologists; (3) early publications on the use of psychological knowledge and techniques in the work of the Stasi; (4) psychologists' and psychotherapists' contributions to “transformation studies” in the early 1990s.

(1) Historical studies of psychology in the GDR: Third‐person reflections.
  Historical reflection on psychology in the GDR began relatively quickly, due in part to the fact that some East German scholars had already acquired skills in historical scholarship. One of them, the above‐mentioned Georg Eckardt, published a paper in 1992 on the role of stage‐managed “controversies” (*Meinungsstreite*) in the politics of East German psychology, based on the example of social psychology at his own university of Jena (Eckardt, 1995). Eckardt went on to supervise a series of theses and dissertations in this topic area, most prominently Kitty Dumont's dissertation on social psychology in the GDR (Dumont, 1999). Parallel to this, Stefan Busse, a student of Manfred Vorwerg in Leipzig, published a paper entitled “Was there a GDR Psychology?” (Busse, 1999), laying the conceptual foundations for a full‐length monograph (Busse, 2004), for which autobiographical interviews were to provide some of the material. To these, I now turn.
(2) Historical studies of psychology in the GDR: First‐person reflections.
  A great number of first‐person reflections by East German psychologists appeared during the 1990s (see, e.g., Sprung, 1992). Richer in reflexive content was a collection of interviews with leading East German psychologists edited by Stefan Busse (1996b), entitled “Psychology under Real Existing Socialism,” based on interviews conducted in 1993 and 1994. Busse's introduction also took a reflexive tack, as he elaborated at length on his own position as interviewer (see also Busse, 1995). Having studied with Manfred Vorwerg, a leading proponent of “Marxist social psychology” in Leipzig, and having established contact in the 1980s with the West German Marxist approach called “Critical Psychology,” which was not well received by his teachers, he acknowledged that his position was hardly neutral.
  As might be expected, Busse's subjects adopted a variety of stances. Friedhart Klix (1996), for instance, claimed that the experimental and mathematical psychology he had established in Berlin was “relatively ideology free,” but he also acknowledged that he told the relevant party officials that high‐quality basic research was needed to improve productivity, which was, of course, an ideological claim. Hacker (1996) faced this dilemma still more squarely since the stated goal of his work in labor and industrial psychology at the TU Dresden was to improve productivity in state‐owned enterprises. Socialist rationality (the idea of “psychology as a productive force”) clashed here with the aim of improving working conditions and thus the self‐satisfaction of workers. The compromise claim that labor psychology was “supportive for personality development” (*persönlichkeitsfördernd*), was accepted by party officials, but Hacker acknowledged that the idea of actually achieving this goal was “a dream”. Other interviewees, such as Gisela Ehrhardt (1996) and Adolf Kossakowsky (1996), who were affiliated with social and educational psychology, claimed to be sincere socialists to the end. They saved themselves from acknowledging their shared responsibility for the failure of the regime by creating, or inventing, a distinction between “real” (meaning ideal) socialism and actual dictatorial conditions. This is ironic, since “Real Existing Socialism” was the term normally used to describe the actual regime.
  In his summary, Busse is unsparing: in his view, the belief that it was possible to be committed both to psychological science and to GDR socialism was a “constructive illusion” (Busse, 1996a, p. 29). But he also points to parallels with West German psychology, claiming that its alleged neutrality masks its social functions—the rationalization of capitalist productivity and the (self‐)optimization of human subjects.
  Hans Dieter Schmidt, longtime professor of developmental psychology at the Humboldt University, entered this discussion with a memoir of his teacher Kurt Gottschaldt, the leading psychologist in East Germany from 1947 to 1962 who never joined the Socialist Unity Party, published in 1992 (Schmidt, 1992). The paper provides valuable material for historical scholarship, but also includes revealing autobiographical remarks. While studying Gottschaldt's skilled efforts to avoid ideological influences, including explicit criticism of Pavlovism in the early 1950s, Schmidt found himself asking “how did he manage that?” and acknowledged in passing that he might ask the same question about his own career. In 1998, Schmidt then published a volume of autobiographical reflections entitled “Texts between Yes and No: Self‐Inquiry of a GDR Psychologist” (Schmidt, 1998), blending the personal, the scientific, and the political (see also Schmidt, 1996). In this work, he attempted to explain or at least render understandable his early loyalty to the GDR, and his later efforts to position himself as a nondogmatic Marxist psychologist in his work on psychological development, via critical debate with “official” Soviet thinkers such as Sergei Rubinstein and Wassily Leontiev. In essence, the narrative is a series of stories of hope and disillusion. He was most rueful about his encounter with party hacks in the pedagogical faculty during the 1980s, revealing the compromises he considered necessary to assure being left alone in his “niche.” In 1989 and 1990 came a burst of explicitly political activity. (Later he held academic power for a time as Dean of the Faculty of Natural Sciences.) He tried quite early to articulate possibilities for political psychology in an address to the first joint congress of the German Psychological Society in Kiel in 1990 (Schmidt, 1991a), in a longer article entitled “Psychology and Politics” written in the same year (Schmidt, 1991b), and also in a report written with others on their investigation of police violence against demonstrators in October 1990. In the autobiographical volume, he openly acknowledged the frustration he felt at the failure of such engagements. His move to autobiography could perhaps be interpreted as a compensatory tactic, a way to help himself work through this frustration.
(3) Stasi‐psychology: scholarship as political intervention.
  In 1990 Jürgen Fuchs, a psychologist trained in Jena and a political dissident who was expelled to the West after a prison sentence, republished protocols of his interrogation by Stasi operatives originally published in 1976 and 1977 (Fuchs, 1976, 1977, 1990). This was an act of revenge, but also a political intervention, intended to expose mechanisms of repression utilizing what the Stasi operatives themselves called “the human factor.” Fuchs' widely noticed publications initiated intensive research and debate on this topic that continues to this day (Maercker & Gieseke, 2021; Maercker & Guski‐Leinwand, 2018; Maercker et al., 2022; Michels & Wieser, 2018; for psychoanalytic reflections on this topic see A. Simon, 2006). Here, I mention only a conference held in the former Stasi headquarters in East Berlin to reflect on the scientific content behind the Stasi's “Operative Psychology.” The proceedings were published in 1995 under the title, “Undermining the Soul” (*Zersetzung der Seele*) (Behnke & Fuchs, 1995). In his opening chapter, co‐editor Klaus Behnke reported on the aims of psychological training offered at the Stasi training academy in Golm, near Potsdam (called “Juristische Hochschule” to disguise its identity) and in the field, including informer selection and training, as well as a detailed listing of psychological tactics employed to disrupt dissident activity, indeed to break dissidents' resolve and perhaps drive them to insanity or even suicide. As chapters by Jürgen Fuchs and Edith Wolf showed, Stasi officers actually studied psychology at the University of Jena in the 1960s and 1970s and later. A student colleague of Fuchs' later became a training officer and instructor in Golm. Thus, a complex picture emerges linking academic psychology and repressive tactics at multiple levels.
  An issue that pervaded the volume was whether it is correct to describe the tactics or techniques actually utilized by the secret police as applications of scientific psychological knowledge, or whether quotations from textbooks by Pavlov, Rubinstein, and Leontjew served as cover for traditional secret police techniques of psychological torture. In an invited commentary (Ash, 1995), I suggested at the time that such legitimatory tactics were typical of the mobilization of psychological knowledge claims in technocratic contexts. More recent work on this topic has emphasized the tension between scientific norms and repressive secret police practices (Schmiedebach, 2021; Michels & Wieser, 2018, Wieser, 2021). Labeling all this “pseudoscience” (as did Richter, 2001) seems unhelpful, even misleading. The ambivalence of such defensive tactics appears obvious on reflection. Do evil regimes require “good” science to function “better,” meaning more efficiently? Do we require Stasi psychology to have been “pseudoscience” to denounce it and the regime more effectively?
(4) “Transitology”.
  The field of “transformation studies” literally exploded onto the scene immediately following unification. The opportunity to study radical social changes happening at such a pace literally on one's own doorstep proved irresistible. In this context I would like to focus on studies in which the author utilized methods or tools of his/her own science to reflect on events and processes in which s/he was a participant, and to ask whether such reflexivity resulted in scientific change. A possible example is work by Hans Dieter Schmidt and West German psychologist Jutta Heckhausen. They began their East‐West research collaboration very soon after unification, and published first results in the proceedings of the congress of the German Society for Psychology in 1992 (Heckhausen & Schmidt, 1992; Schmidt & Heckhausen, 1992, 1994). Schmidt later wrote that they were nearly alone at first, but a year later nearly 30 other papers were presented in the same topic area. They found that their subjects experienced pressure for a stronger orientation toward “material values,” which they compensated with strategies ranging from “coping” to “defending.” “Where acculturation pressures and burdens appeared,” the result was “massive identity conflicts and endangerment” due to “a one‐sided adaptation of East to West, … indicated by resignation (and) uncertainty in dealing with intensified competition.” Perhaps most alarming was their claim that “Behind these identification conflicts lie latent aggression potential.” They recommended that psychologists pay more attention to these symptoms in the interest of “prophylaxis and therapeutic assistance,” but also that they go beyond such efforts and engage directly in “policy advice.”

Hans‐Joachim Maaz also diagnosed similar psychosocial problems, while engaging not in policy advice, but in social commentary at the moment of unification. A medically trained psychotherapist, Maaz had been head of the Psychotherapeutic Clinic in Halle, supported by the Lutheran Church, since 1980, where he had worked from this relatively safe “niche” toward instituting psychoanalytic and body‐therapeutic approaches to clinical work, sometimes using underground tactics. Like Schmidt, Maaz broke out early, but he did so in a very different way, becoming a best‐selling author with two books of popular psychological diagnosis, “The Emotional Blockade” (*Der Gefühlsstau*) and “The Fallen People” (*Das gestürzte Volk*) published in 1990 and 1991, respectively (Maaz, 1990, 1991). Apparently, these best‐selling writings fulfilled a need to explain the obvious disappointment of many East Germans with their lives in unified Germany, and perhaps also West German demand for texts that would explain or interpret these new Germans in understandable language. There is nothing scientific about these books; although Maaz claimed to base his diagnoses on his clinical work as well as on numerous conversations with West German colleagues, he never stated exactly how he came to his very broad, often overdrawn conclusions about “the East Germans” and their mentality. I include him here because these books had, for a time, far higher public impact than anything academic psychologists or other human scientists wrote, and focus here on Maaz's second book, “The Fallen People” (Maaz, 1991).

Maaz claims that the personality structure East Germans needed to develop to survive in and adapt to a repressive regime was deeply ambivalent: dependence on a caring socialist “mother,” who provided employment and social security predominated. Unfreedom was accepted in exchange, and compensated by low‐level avoidance strategies aimed at establishing “niches” supported by localized familial and other, constantly shifting networks. The mass demonstrations of 1989 provided welcome release, as safety in numbers assured freedom from fear. The fall of the wall released an emotional rush of suppressed desires for freedom, but also for travel and material goods, suddenly and seemingly easily fulfilled. “The East Germans” chose unification willingly on the basis of phantasies about the freedom and prosperity it would bring, with no loss of social security. Necessary lies of West German politicians, addressed to Eastern and Western Germans, fed this dynamic (“blooming landscapes,” “no new taxes”; “no one will be worse off”). In reality, Maaz wrote, East Germans had “voted for their own defeat.” In such a situation disappointment was inevitable. The reaction was a new dynamic of submission to “mother Deutschmark.” This was met with complete incomprehension by West Germans, frustrated in their turn by East Germans' seemingly submissive behavior, and what was perceived to be their resentful and ungrateful attitudes.

Perhaps most intriguing was Maaz's claim that this dynamic was actually mutual—West Germans, after all, had their own carefully suppressed anxieties and frustrations, about which West German colleagues made sure to inform Maaz during his conversations with them. Needed, he argued, was “a third way” or even “a third Germany,” in which both “sides” could meet and exchange views without fear. Maaz's inclusion of a rollicking account of his own roller‐coaster emotions following the success of *Der Gefühlsstau* in a forward to “The Fallen People,” and his occasional use of “we” rather than the third person for “the East” made his writing lively, and also played to the (West) German demand, fashionable then and now, that authors “put themselves into the story.” Whether or not this was a genuine case of scientific innovation achieved through reflexivity is another matter entirely.

## 5 CONCLUSIONS

As I stated at the outset, this is a preliminary study. I have tried to sketch some examples of how political regime change affected psychology in Eastern Germany after the fall of Communism. In part two of my remarks, I showed how psychologists became objects of higher education policy decisions, and also how institutional arrangements in psychology were completely restructured at East German universities following German unification. The autobiographical accounts discussed in part three might be considered examples of how some psychologists tried to recover, or reconstruct, their identities in response to (or: in self‐defense against) policy decisions made by others, how other psychologists addressed the political role of psychological knowledge in maintaining a dictatorial regime, and how some human scientists attempted to influence policy debates as well as public discourse about East Germany. The results of these efforts at the time appear to have been mixed, at best. The long‐term impact of academic psychologists' contributions to “transitology” is unclear and remains to be studied in more detail.

Much of the historical scholarship I have mentioned ought to be of interest to historians of science more broadly, but is not likely to be noticed outside psychology. Stefan Busse's approach was an attempt to incorporate reflexivity into historical scholarship and was thus potentially innovative, but this approach does not seem to have attracted many followers compared with more recent work intended to advance ethically informed self‐reflection within psychology (e.g., Guski‐Leinwand & Nussmann, 2021; Strauß et al., 2022). Whether social‐scientific transformation studies have ever done more than provide seismographic markers of changing social status and attitudes as they take place is still an open question, one that has taken on new meaning in the face of the vast refugee movements now happening in response to the current war in Ukraine.

## Data Availability

My data are available from the following personal website: https://www.univie.ac.at/Geschichte/_ash.html.

## Endnotes

1 All of these decision points were determined largely by domestic policy considerations. Equally important was the so‐called “two plus four” agreement negotiated parallel to the Unification Treaty and signed on September 12, 1990, in which the two German states, the United States, Britain, France, and the still‐existing Soviet Union assured the withdrawal of foreign troops and thus established full sovereignty for the new German state. See Sarotte (2014) and Lillteicher (2021).

2 At this point a personal note might be in order. As a PhD student working in Germany on the history of Gestalt psychology in the late 1970s and early 1980s, I was invited to speak at philosophy and psychology seminars in East Berlin, participated in the International Congress of Psychology in Leipzig in 1980, and was later among the few American researchers allowed to work in East German archives. During this period, I came to know the small community of East German historians of psychology and maintained friendships with some of them that continued into the 1990s and later. I was also in Berlin regularly from the date of unification to 1997, witnessed some of the upheavals described here first hand, and learned more from conversations with colleagues.
